# Supplementary material for: Acteoside Ameliorates Hepatic Steatosis and Liver Injury in MASLD Mice Through Activation of PINK1/Parkin-Related Mitophagy Markers
Source: Nutrients. 2025 Dec 29;18(1):118. doi: 10.3390/nu18010118 (PMC12787717; doi:10.3390/nu18010118)
Supplement: Supplementary file 1 [file nutrients-18-00118-s001.zip › nutrients-3996824-supplementary.pdf]

# Supplementary Materials

This supplementary materials file contains the following contents:

| | |
|---|---|
| Supplementary Tables | Table S1 to S9 |
| Supplementary Figures | Figure S1 to S7 |

**Table S1: Body weight of mice during weeks 0–7.**

| Group | 0 week | 1 week | 2 week | 3 week | 4 week | 5 week | 6 week | 7 week |
|---|---|---|---|---|---|---|---|---|
| Control | 20.59±0.60 | 22.05±0.87 | 21.84±1.18 | 23.27±0.96 | 23.40±0.82 | 23.93±1.02 | 24.89±0.99 | 25.46±0.91 |
| Model | 20.59±0.68 | 22.67±1.03 | 23.60±1.22 | 25.80±1.26 | 26.39±1.32 | 27.48±1.44 | 28.81±1.36 | 30.10±2.04 |
| ACT(120mg/kg) | 20.29±0.75 | 22.05±0.87 | 22.71±1.25 | 24.75±1.29 | 25.63±1.35 | 26.60±1.46 | 28.02±1.21 | 28.96±1.44 |
| ACT(60mg/kg) | 20.35±0.90 | 21.69±1.25 | 22.51±1.18 | 24.08±1.25 | 25.21±1.27 | 26.24±1.76 | 28.46±1.76 | 29.09±1.90 |
| ACT(30mg/kg) | 20.25±0.65 | 21.47±1.61 | 23.78±1.25 | 25.59±1.41 | 26.47±1.50 | 26.97±1.21 | 28.51±1.57 | 29.91±2.31 |
| RSF (3mg/kg) | 20.57±0.66 | 22.28±1.42 | 23.58±1.06 | 25.12±1.33 | 25.88±1.43 | 26.76±1.08 | 28.22±1.57 | 28.63±1.41 |

Data are presented as mean ± SEM (n = 8).

**Table S2: Body weight of mice during weeks 7-14**

| Group | 8 week | 9 week | 10 week | 11 week | 12 week | 13 week | 14 week |
|---|---|---|---|---|---|---|---|
| Control | 25.36±1.17 | 26.01±1.85 | 25.81±1.28 | 26.31±1.24 | 26.25±1.52 | 26.60±1.30 | 26.25±1.47 |
| Model | 30.86±2.46 | 32.05±2.58 | 32.95±2.78 | 34.16±2.58 | 34.74±2.55 | 35.45±2.67 | 36.41±2.14 |
| ACT(120mg/kg) | 29.95±1.34 | 30.71±1.59 | 31.11±1.32 | 31.34±1.30 | 31.73±1.45 | 31.88±1.39 | 31.91±1.41 |
| ACT(60mg/kg) | 30.17±2.12 | 30.91±2.15 | 31.75±2.16 | 32.11±2.25 | 33.42±2.39 | 33.94±2.17 | 34.42±2.09 |
| ACT(30mg/kg) | 30.19±2.66 | 31.06±2.06 | 32.40±2.23 | 32.27±2.17 | 33.87±2.17 | 34.21±2.08 | 34.74±1.98 |
| RSF (3mg/kg) | 29.13±1.64 | 29.83±1.83 | 30.54±1.58 | 31.37±1.73 | 32.21±1.44 | 32.44±1.62 | 31.94±1.36 |

Data are presented as mean ± SEM (n = 8).

**Table S3: Effects of ACT on body weight, liver weight, liver index, liver weight, and serum ALT and AST levels in mice**

| Group | body weight /g | liver weight /g | liver index /% | AST/U·L$^{-1}$ | ALT/U·L$^{-1}$ |
|---|---|---|---|---|---|
| Control | 26.25±1.47** | 0.95±0.07** | 2.79±0.11** | 98.8±9.94** | 31.98±4.93** |
| Model | 36.41±2.14 | 1.34±0.14 | 3.96±0.23 | 216.01±29.70 | 59.61±7.59 |
| ACT(120mg/kg) | 31.91±1.41** | 1.10±0.03* | 3.28±0.12** | 111.40±13.30** | 20.88±0.79** |
| ACT(60mg/kg) | 34.42±2.09* | 1.21±0.07* | 3.68±0.34 | 121.03±12.30** | 33.65±3.46* |
| ACT(30mg/kg) | 34.74±1.98* | 1.28±0.15 | 3.72±0.39 | 136.26±8.93** | 22.05±2.19** |
| RSF (3mg/kg) | 31.94±1.36** | 1.03±0.09** | 3.29±0.36* | 133.41±5.19** | 18.27±2.84** |

Data are presented as mean ± SEM (n = 8). *$P$ < 0.05, **$P$ < 0.01, compared with Model group.

**Table S4: Effects of ACT on serum TC, HDL-C, TG, and LDL-C levels in mice**

| Group | TG/mmol·L$^{-1}$ | TC/mmol·L$^{-1}$ | HDL-C/mmol·L$^{-1}$ | LDL-C/mmol·L$^{-1}$ |
|---|---|---|---|---|
| Control | 0.81±0.05** | 2.79±0.07** | 1.89±0.09** | 0.36±0.07** |
| Model | 1.10±0.11 | 5.34±0.34 | 0.73±0.16 | 0.76±0.12 |
| ACT(120mg/kg) | 0.71±0.07** | 4.30±0.15* | 1.91±0.15** | 0.58±0.05* |
| ACT(60mg/kg) | 1.03±0.14 | 4.73±0.16* | 1.40±0.20* | 0.70±0.09 |
| ACT(30mg/kg) | 1.04±0.07 | 5.33±0.25 | 1.27±0.21* | 0.73±0.09 |
| RSF (3mg/kg) | 0.71±0.09** | 4.11±0.43* | 1.77±0.17** | 0.40±0.07** |

Data are presented as mean ± SEM (n = 8). *$P$ < 0.05, **$P$ < 0.01, compared with Model group.

**Table S5: Effects of ACT on FBG, FINS, HOMA-IR index in mice**

| Group | FBG/ mmol·L$^{-1}$ | FINS/μIU·L$^{-1}$ | HOMA-IR |
|---|---|---|---|
| Control | 6.10±0.60** | 8.66±0.55** | 2.35±0.26** |
| Model | 9.89±0.77 | 12.73±0.98 | 5.60±0.68 |
| ACT(120mg/kg) | 7.88±0.70* | 9.75±0.47* | 3.41±0.32** |
| ACT(60mg/kg) | 8.88±0.80 | 10.18±0.50* | 4.01±0.40* |
| ACT(30mg/kg) | 9.55±0.57 | 10.43±0.64* | 4.43±0.45* |
| RSF (3mg/kg) | 8.18±0.97* | 9.60±0.64* | 3.48±0.43** |

Data are presented as mean ± SEM (n = 8). *$P$ < 0.05, **$P$ < 0.01, compared with Model group.

**Table S6: Effects of ACT on SOD activity and MDA and GSH-PX levels in mice**

| Group | SOD/U·mL$^{-1}$ | GSH-Px/U·mgprot$^{-1}$ | MDA/U·mL$^{-1}$ |
|---|---|---|---|
| Control | 268.00±14.61** | 6811.08±833.87** | 223.74±12.03** |
| Model | 194.67±11.62 | 4601.78±1036.13 | 261.52±10.72 |
| ACT(120mg/kg) | 235.90±6.63* | 6965.07±719.44** | 222.56±12.21** |
| ACT(60mg/kg) | 228.57±9.75* | 6516.29±1195.79* | 230.03±12.67* |
| ACT(30mg/kg) | 215.69±11.99 | 5873.61±869.92 | 233.08±6.15* |
| RSF (3mg/kg) | 233.79±8.33** | 6883.92±1328.80** | 219.79±19.14** |

Data are presented as mean ± SEM (n = 8).*$P$ < 0.05, **$P$ < 0.01, compared with Model group.

**Table S7: Effects of ACT on TNF-$\alpha$, IL-1β, and IL-6 levels in liver tissue of mice**

| Group | TNF-α/pg·ug$^{-1}$ | IL-1β/pg·ug$^{-1}$ | IL-6/pg·ug$^{-1}$ |
|---|---|---|---|
| Control | 3.71±0.25** | 2.11±0.37** | 8.56±0.34** |
| Model | 7.69±0.47 | 3.81±0.37 | 18.48±1.19 |
| ACT(120mg/kg) | 5.43±0.57** | 2.25±0.27** | 8.79±0.18** |
| ACT(60mg/kg) | 6.74±0.25* | 3.10±0.51* | 9.65±0.44** |
| ACT(30mg/kg) | 7.03±0.52 | 3.51±0.34 | 12.17±0.38* |
| RSF (3mg/kg) | 4.78±0.64** | 2.07±0.25** | 8.35±0.35** |

Data are presented as mean ± SEM (n = 8). *$P$ < 0.05, **$P$ < 0.01, compared with Model group.

**Table S8: Quantification of PINK1, Parkin, LC3, p62, and p-p62 protein expression by western blot**

| Group | Parkin | PINK1 | p-P62 | P62 | LC3II/LC3I |
|---|---|---|---|---|---|
| Control | 0.47±0.08** | 0.72±0.09** | 0.16±0.04*** | 0.05±0.01** | 0.81±0.06** |
| Model | 0.04±0.01 | 0.07±0.03 | 0.69±0.06 | 0.64±0.06 | 0.36±0.01 |
| ACT(120mg/kg) | 0.27±0.06** | 0.48±0.05** | 0.26±0.04** | 0.15±0.04** | 0.66±0.01** |
| ACT(60mg/kg) | 0.17±0.04* | 0.28±0.06* | 0.36±0.03** | 0.24±0.03** | 0.54±0.04* |
| ACT(30mg/kg) | 0.06±0.01 | 0.12±0.04 | 0.52±0.06* | 0.46±0.06* | 0.43±0.04 |
| RSF (3mg/kg) | 0.15±0.07* | 0.24±0.09* | 0.37±0.04** | 0.28±0.07** | 0.52±0.02* |

Data are presented as mean ± SEM (n = 3). *$P$ < 0.05, **$P$ < 0.01, ***$P$ < 0.001, compared with Model group.

**Table S9: Quantification of PINK1, Parkin, LC3, p62, and p-p62 immunoreactivity**

| Group | Parkin | PINK1 | p-P62 | P62 | LC3II/LC3I |
|---|---|---|---|---|---|
| Control | 16.52±3.30*** | 13.12±6.31** | 0.75±0.31*** | 0.55±0.31*** | 7.98±3.81** |
| Model | 0.56±0.12 | 0.74±0.13 | 13.10±1.33 | 11.40±2.18 | 0.98±0.12 |
| ACT(120mg/kg) | 13.44±1.95*** | 6.89±3.89** | 3.08±1.19** | 1.81±1.07*** | 6.41±1.02** |
| ACT(60mg/kg) | 9.37±1.86*** | 6.32±4.23* | 8.86±2.54* | 6.08±1.49** | 5.81±2.07** |
| ACT(30mg/kg) | 2.17±0.78* | 1.57±0.21 | 10.32±3.59* | 9.84±1.89 | 2.71±1.23* |
| RSF (3mg/kg) | 6.92±1.94** | 3.86±1.16* | 7.69±0.96* | 4.36±0.94** | 4.35±0.19** |

Data are presented as mean ± SEM (n = 3). *$P$ < 0.05, **$P$ < 0.01, ***$P$ < 0.001, compared with Model group.

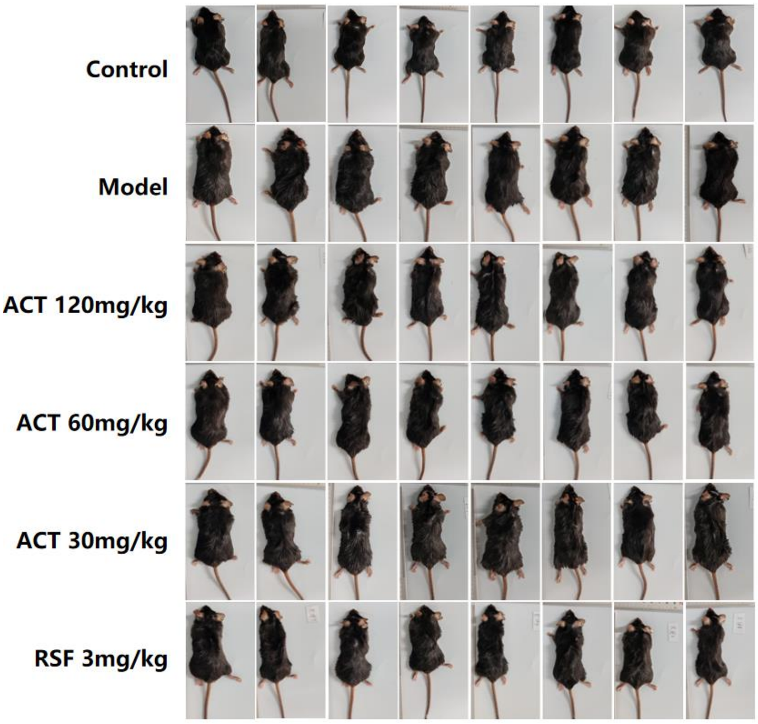


**Figure S1. Body weight of mice in each group at the end of the experiment**

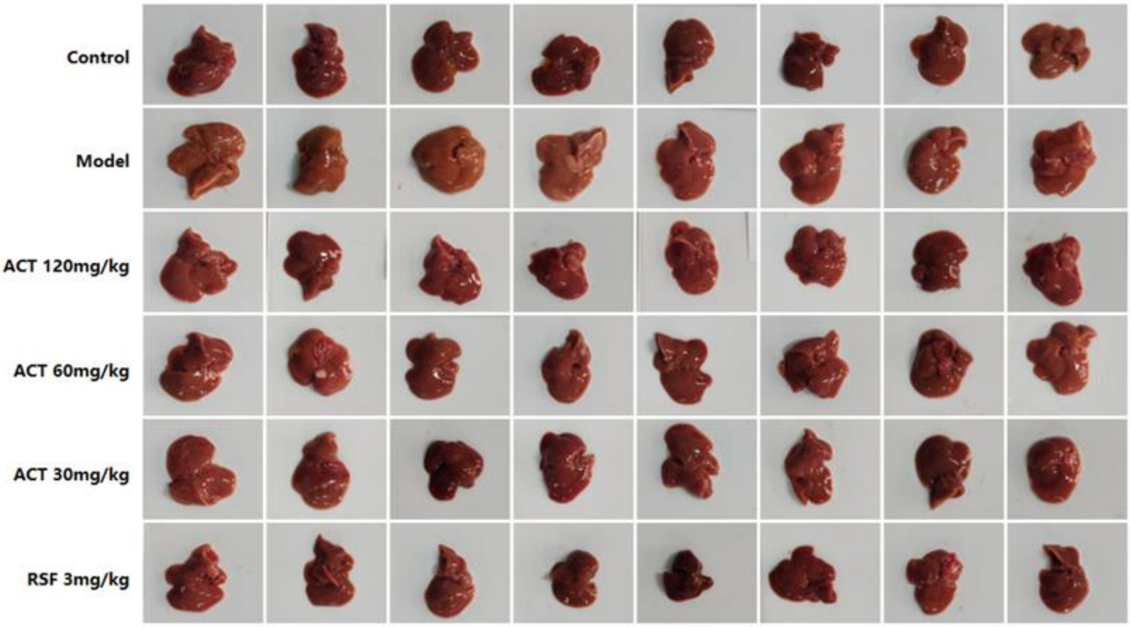


**Figure S2. Gross morphology of liver tissues from each group at the end of the experiment**

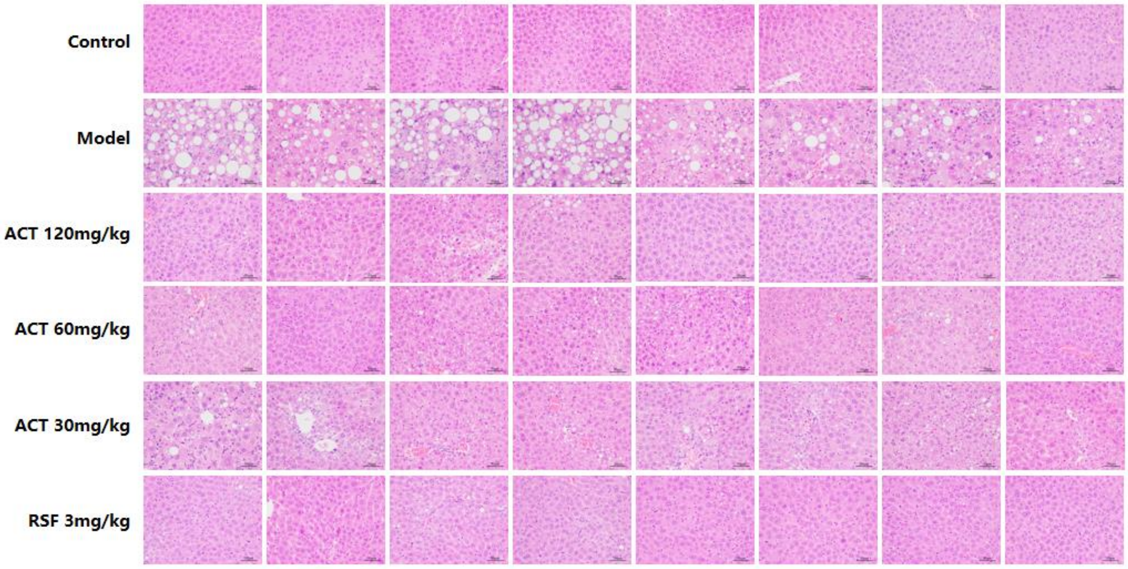


**Figure S3. Representative photomicrographs of H&E-stained liver sections**

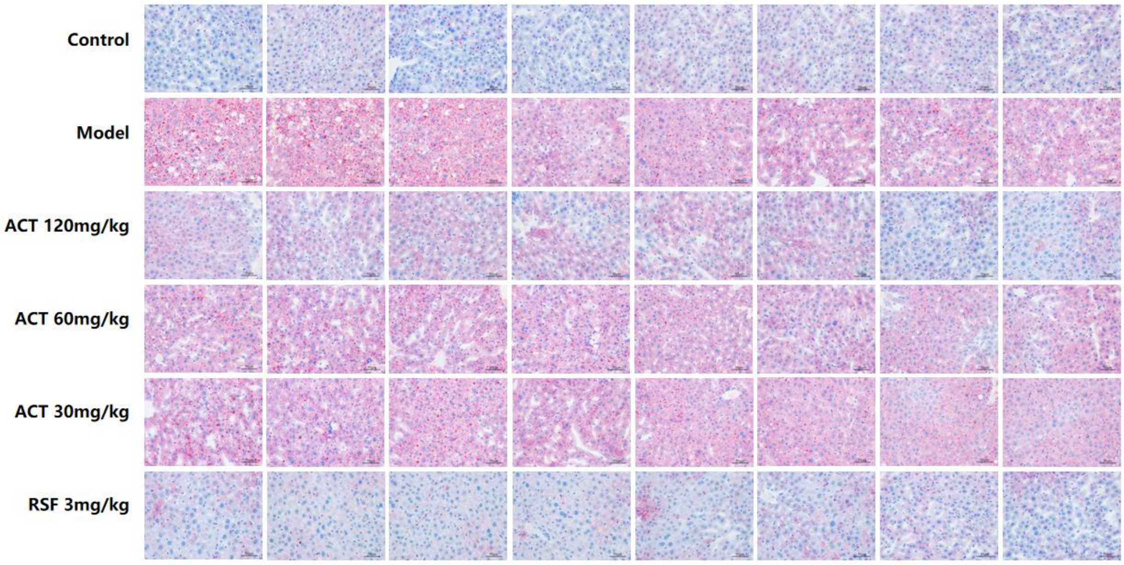


**Figure S4. Representative photomicrographs of Oil Red O-stained liver sections**

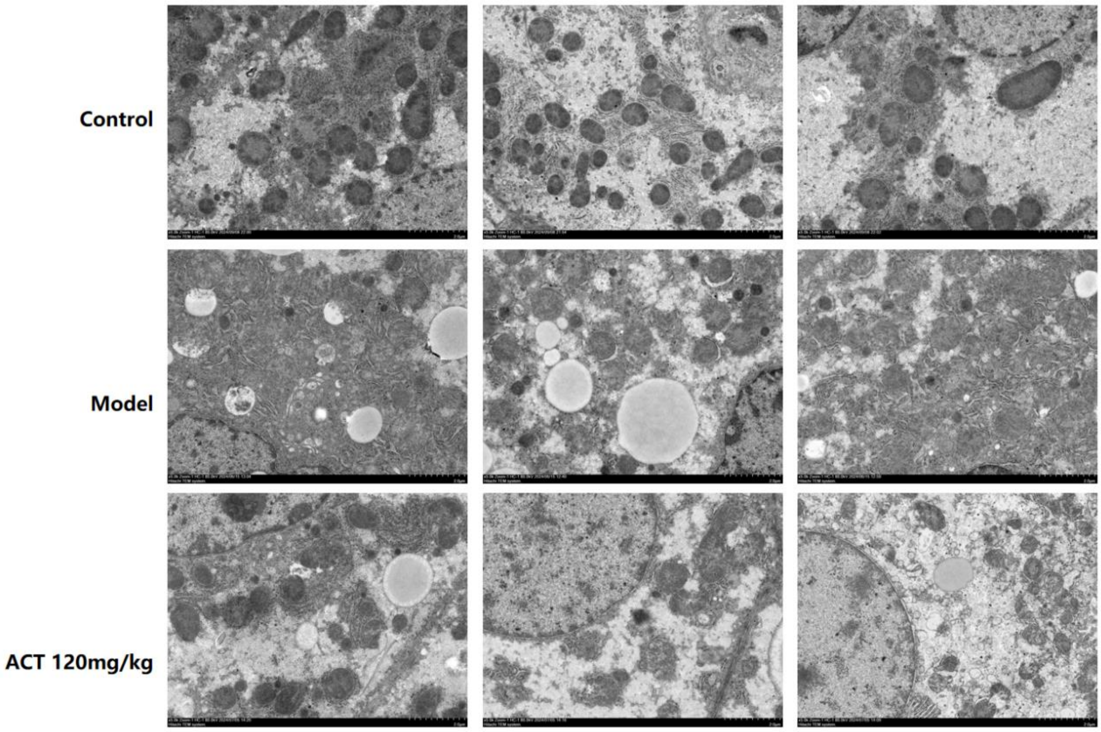


**Figure S5. Representative transmission electron microscopy (TEM) images of liver sections**

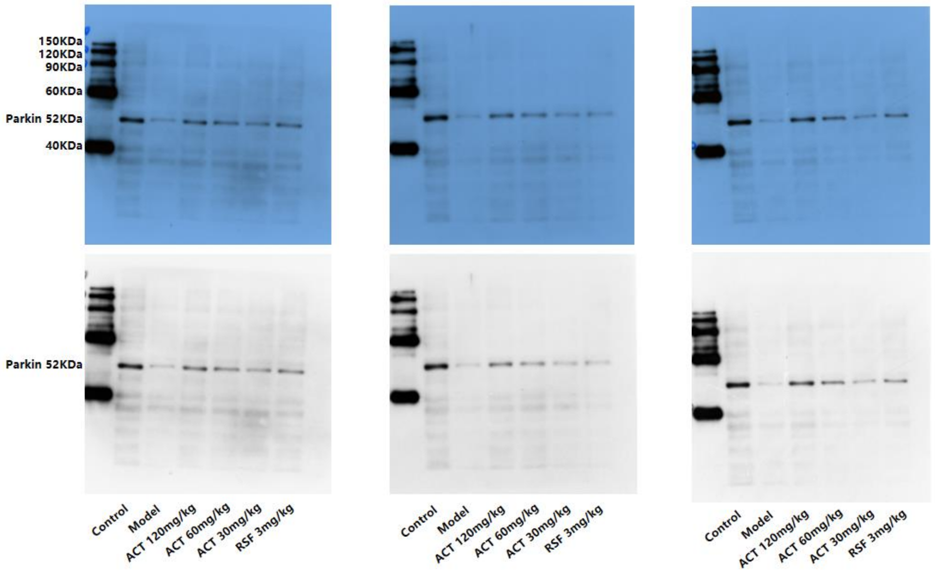
150KDa
120KDa
90KDa
60KDa
Parkin 52KDa
40KDa
Parkin 52KDa
Control
Model
ACT 120mg/kg
ACT 60mg/kg
ACT 30mg/kg
RSF 3mg/kg

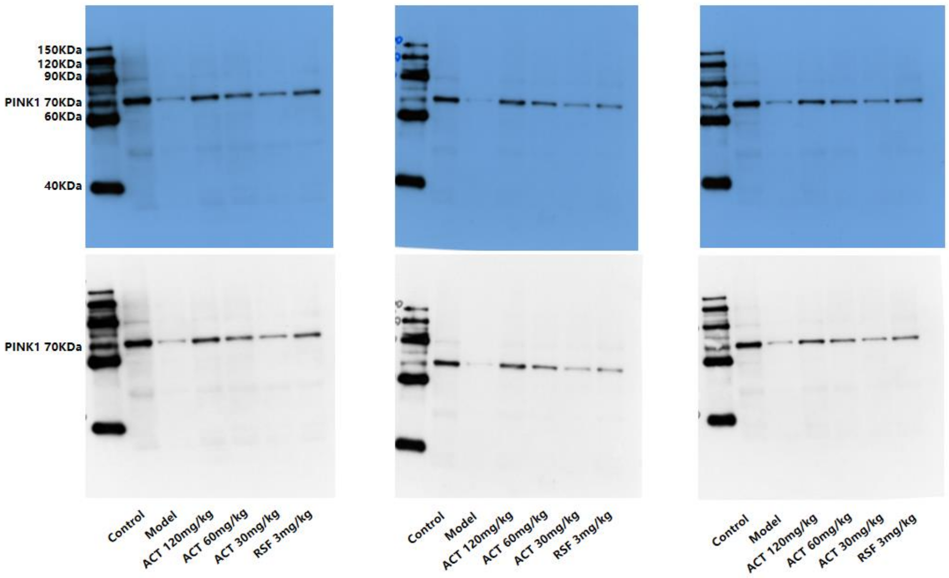
150KDa
120KDa
90KDa
PINK1 70KDa
60KDa
40KDa
PINK1 70KDa
Control
Model
ACT 120mg/kg
ACT 60mg/kg
ACT 30mg/kg
RSF 3mg/kg

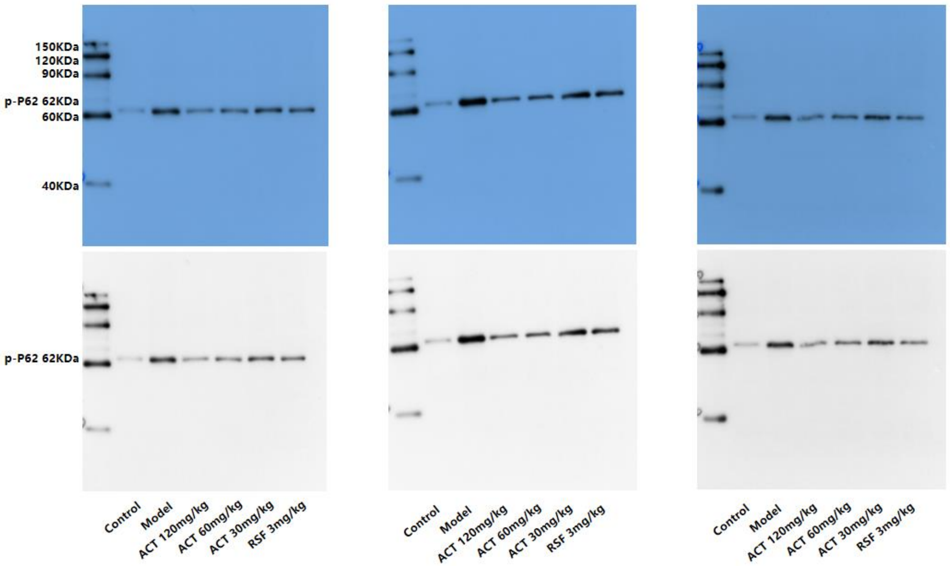
150KDa
120KDa
90KDa
p-P62 62KDa
60KDa
40KDa
p-P62 62KDa
Control
Model
ACT 120mg/kg
ACT 60mg/kg
ACT 30mg/kg
RSF 3mg/kg

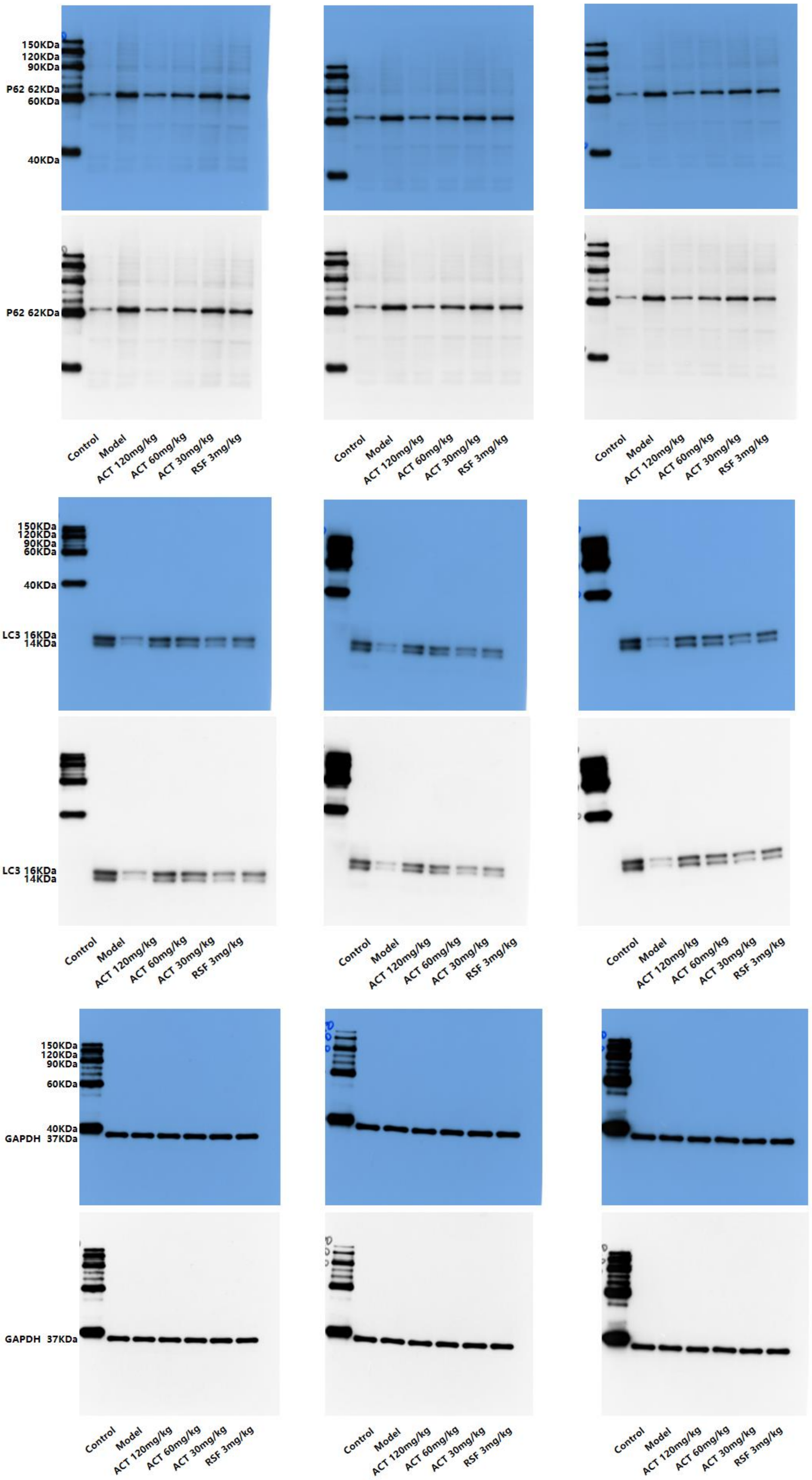


**Figure S6. Western blot analysis of PINK1, Parkin, LC3, p62, and p-p62 protein expression in mouse liver tissues**

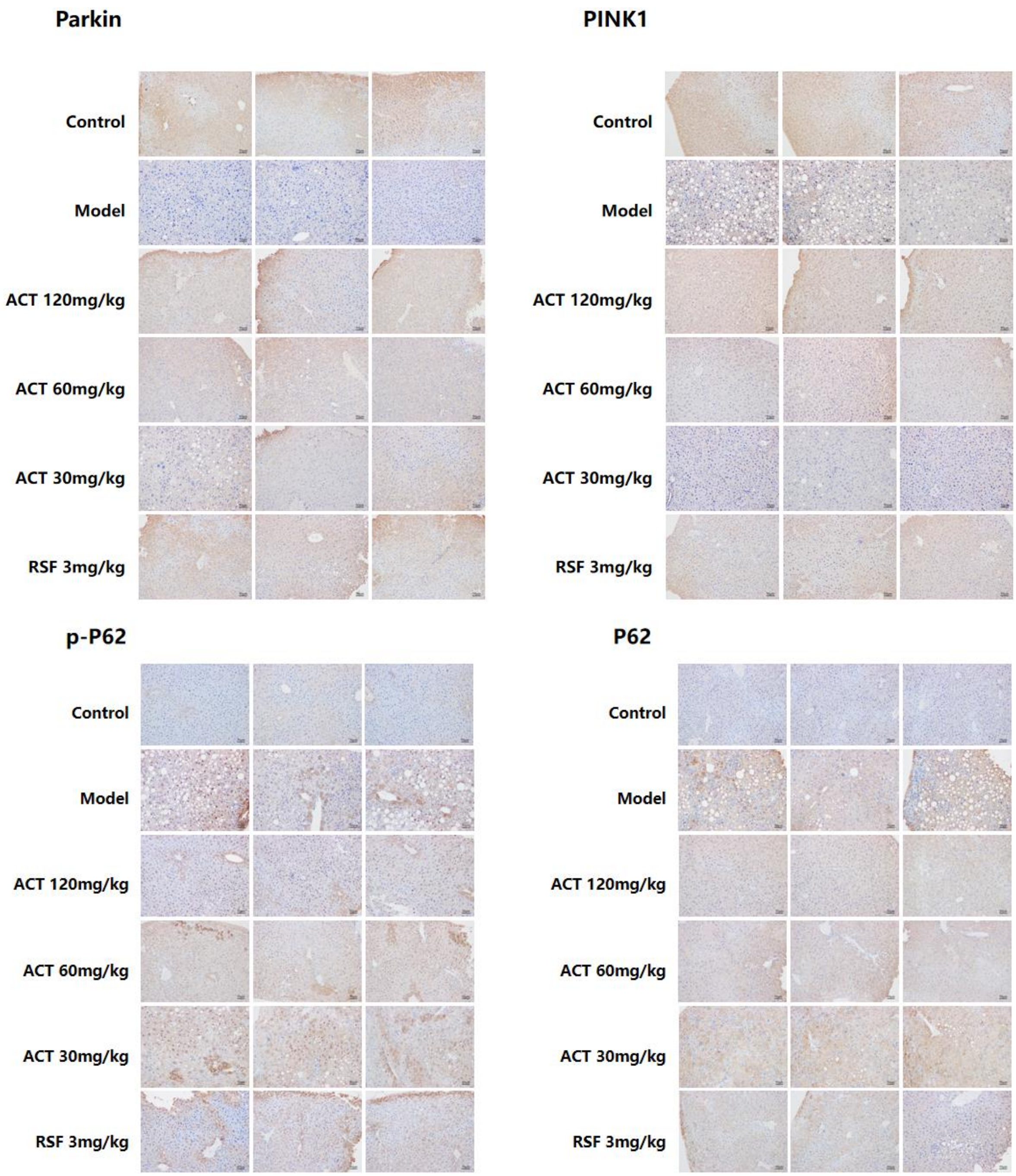
Parkin
Control
Model
ACT 120mg/kg
ACT 60mg/kg
ACT 30mg/kg
RSF 3mg/kg
PINK1
Control
Model
ACT 120mg/kg
ACT 60mg/kg
ACT 30mg/kg
RSF 3mg/kg
p-P62
Control
Model
ACT 120mg/kg
ACT 60mg/kg
ACT 30mg/kg
RSF 3mg/kg
P62
Control
Model
ACT 120mg/kg
ACT 60mg/kg
ACT 30mg/kg
RSF 3mg/kg

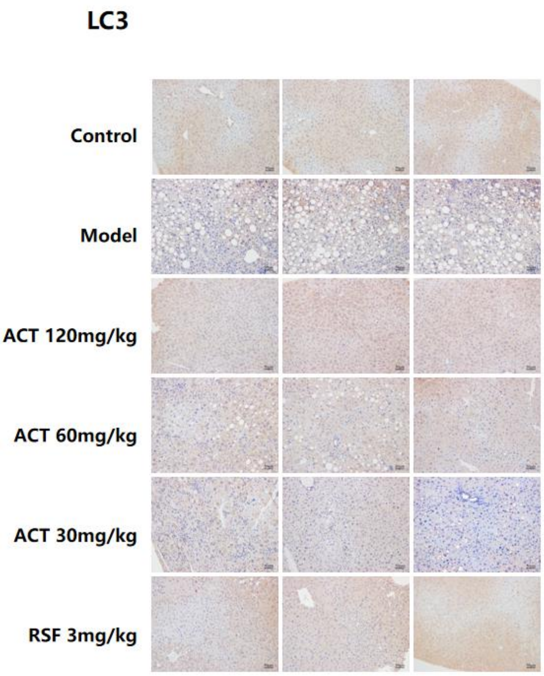


**Figure S7. Immunohistochemical staining of PINK1, Parkin, LC3, p62, and p-p62 in mouse liver tissues.**
